# Enhancing lower limb and core muscle activation with blood flow restriction training: a randomized crossover study on high-intensity squat exercises

**DOI:** 10.3389/fphys.2024.1436441

**Published:** 2024-08-05

**Authors:** Sunyoumeng Zhuan, Yutong Zhu, Jingyi Zhou, Senlin Lei, Xin Wang, Juan Li

**Affiliations:** ^1^ Linyi Vocational College of Science and Technology, Linyi, Shandong, China; ^2^ School of Physical Education, China University of Mining and Technology, Xuzhou, Jiangsu, China; ^3^ Hebei Oriental University, School of Humanities, Langfang, Hebei, China; ^4^ School of Physical Education and Sports, Beijing Normal University, Beijing, China; ^5^ School of Art, Wuhan Sports University, Wuhan, Hubei, China; ^6^ School of Physical Education, Zhengzhou University, Zhengzhou, Henan, China; ^7^ School of Nursing, Shandong First Medical University & Shandong Academy of Medical Sciences, Taian, Shandong, China

**Keywords:** blood flow restriction training, squat, lower limb muscles, core muscles, degree of muscle activation, rating of perceived exertion

## Abstract

**Objective:**

The primary objective of this study was to assess the impact of high-intensity deep squat training integrated with various blood flow restriction (BFR) modalities on the activation of lower limb and core muscles.

**Methods:**

A randomized, self-controlled crossover experimental design was employed with 12 participants. The exercise protocol consisted of squat training at 75% of one-repetition maximum (1RM), performed in 3 sets of 8 repetitions with a 2-min inter-set rest period. This was conducted under four distinct BFR conditions: continuous low BFR (T1), intermittent medium BFR (T2), intermittent high BFR (T3), and a non-restricted control (C). Surface electromyography (EMG) was utilized to collect EMG signals from the target muscles during the BFR and squat training sessions. The root mean square (RMS) amplitude standard values were calculated for each squat set to quantify muscle activation levels, with these values expressed as a percentage of the maximum voluntary contraction (%MVC). Rating of Perceived Exertion was evaluated after each squat set, and leg circumference measurements were taken.

**Results:**

1) During the first two sets of deep squats, the %MVC of the vastus lateralis and vastus medialis in all compression groups was significantly higher than that in the control group (p < 0.05). Furthermore, in the first set, the %MVC of the vastus lateralis in Group T3 was significantly higher than in Group T2 (p < 0.05). In the third set, the %MVC of the vastus medialis in Groups T1 and T3 was significantly lower than in the first two sets (p < 0.05). 2) Group T1 showed an increased activation of the biceps femoris and semitendinosus muscles in the second and third sets, with %MVC values significantly greater than in the first set (p < 0.05). Group T2 only showed an increase in biceps femoris activation in the third set (p < 0.05). Group T3 significantly increased the activation of the biceps femoris and semitendinosus muscles only in the first set (p < 0.05). 3) No significant differences were observed in the changes of rectus abdominis %MVC among the groups (p > 0.05). In the first set, Group T3’s erector spinae %MVC was significantly higher than the control group’s; in the second set, it was significantly higher than both Group T2 and the control group’s (p < 0.05). 4) After training, a significant increase in thigh circumference was observed in all groups compared to before training (p < 0.05). 5) For RPE values, Group T2’s post-squat values were significantly higher than the control group’s after all three sets (p < 0.05). Group T1’s RPE values were also significantly higher than the control group’s after the third set (p < 0.05). Groups T1, T2, and C all had significantly higher RPE values in the second and third sets compared to the first set (p < 0.05).

**Conclusion:**

All BFR modalities significantly enhanced the activation level of the anterior thigh muscles, with the continuous low BFR mode demonstrating a more stable effect. No significant differences were found in the activation level of the rectus abdominis among the groups. However, the intermittent high BFR mode was the most effective in increasing the activation level of the erector spinae muscles. While BFR did not further augment leg circumference changes, it did elevate subjective fatigue levels. The RPE was lowest during squatting under the intermittent high BFR condition.

## Introduction

Blood Flow Restriction (BFR) is a technique that involves the use of cuffs applied proximally on the limbs to restrict venous blood return during physical activity, thereby diminishing arterial blood flow to the muscles without completely preventing it. This method is designed to enhance the metabolic stress response, surpassing that of traditional training approaches (Jia et al., 2019a; Lin et al., 2023). In recent years, BFR has risen in prominence across competitive sports, physical conditioning, and medical rehabilitation settings due to its efficacy in achieving training outcomes similar to those of high-intensity exercises, even when combined with low-intensity efforts (Shen et al., 2022). BFR has been shown to significantly improve muscle fitness parameters, such as strength, quality, and functionality, when compared to non-BFR training methods (Shen et al., 2022).

The heightened effectiveness of BFR can be ascribed to several mechanisms. Notably, BFR prompts an increased reliance on anaerobic metabolism, which can rapidly fatigue type I muscle fibers, necessitating the recruitment of high-threshold type II muscle fibers (Manini et al., 2011). Additionally, the pressure applied can activate afferent nerve centers, initiating a stress response cascade that may lead to the inhibition of a motor neurons (Yasuda et al., 2006). This results in a greater recruitment of muscle fibers to maintain force production and mechanical output, enhancing muscle activation and adaptability in exercise participants.

Despite the established benefits, there are limitations in the current BFR modalities, with a dearth of research in certain areas. Prevalent research tends to focus on low-intensity (20%–30% 1RM) combined with medium to high pressure (200–300 mmHg) (Liang et al., 2020), medium-intensity (30%–50% 1RM) with low to moderate pressure (100–200 mmHg) (Suga et al., 1985), or high-intensity (75% 1RM) with low pressure (100–150 mmHg) (Yuan, 2018; Lu et al., 2020; Zheng and Zhou, 2021). There is a scarcity of research on high-pressure and high-resistance modes, possibly due to safety concerns regarding potential injuries to cardiovascular function and muscles from sustained high-pressure training (Jia et al., 2019b). However, some studies have begun to explore the application of high BFR in moderate and high-intensity resistance training by varying the restriction mode, reporting improvements in muscle size and strength (Davids et al., 2021; Torma et al., 2021). These studies, however, have not fully elucidated the impact on neuromuscular adaptations during training, particularly concerning changes in muscle electrophysiological signals.

In light of these findings, the present study introduces an intermittent compression intervention to assess the effects of high-intensity squat training with block pressures on the activation levels of the lower limbs and core muscles. The study also incorporates the subjective fatigue index as a measure to evaluate the practicality of this combined training approach. The revised aim of this study is to quantify the effects of high-intensity squat training integrated with specific BFR modalities—continuous low-pressure BFR (T1), intermittent medium-pressure BFR (T2), and intermittent high-pressure BFR (T3)—on the following specific metrics: Muscle activation levels of targeted lower limb muscles (e.g., quadriceps, hamstrings) and core muscles (e.g., rectus abdominis, erector spinae) as measured by Surface Electromyography (EMG); Changes in muscle girth to evaluate muscle volume and potential congestion; Subjective fatigue levels assessed using the RPE scale. We propose two hypotheses regarding the integration of BFR with high-intensity squat training: Hypothesis 1: The combination of high-intensity squat training with various BFR modalities will augment the activation of the lower extremity and core muscles. Hypothesis 2: BFR training will attenuate subjective perceptions of fatigue in comparison to unrestricted training protocols.

## Objective and methods

The experimental study was conducted within the Physical Fitness Room at the School of Physical Education, Zhengzhou University, spanning October to November 2022. A randomized cross-control design coupled with a self-controlled methodology was implemented. Participant recruitment targeted students from the same institution, with stringent inclusion and exclusion criteria applied to an initial pool of 28 candidates. This rigorous selection process culminated in the inclusion of 12 students who met the criteria, thereby comprising the final participant group for the study. Table 1 provides an overview of the participants’ basic information. To mitigate subjective bias inherent in the study’s design, participants were not informed of the true purpose of the experiment until its conclusion. Instead, they were informed of a purported objective, which aimed to assess the impact of pressure stimulation during squat training on muscle training efficacy. The selection of male participants was intentional, considering the potential confounding effects of hormonal fluctuations and physiological differences between sexes that could influence muscle activation and training outcomes. This selection criterion was implemented to maintain consistency in the study’s physiological measurements and to reduce variability that could obscure the effects of the BFR modalities on muscle activation.

**TABLE 1:** Basic information of the subjects.

Age	Height(cm)	Weight (kg)	Left leg circumference(cm)	Right leg circumference (cm)	Squat 1RM (kg)
23 ± 2	173.10 ± 5.95	79.15 ± 5.51	56.02 ± 1.42	57.24 ± 2.21	114.00 ± 21.09

Inclusion criteria encompassed: 1) a minimum of 3 years’ experience in resistance training, 2) demonstrated proficiency in performing squat exercises, and 3) the capability to execute squats at a load of at least 1.2 times their body weight, as verified by one-repetition maximum (1RM) testing.

Exclusion criteria were as follows: 1) a diagnosis of lumbar disc degeneration, 2) any history of lower limb muscle or bone injuries, and 3) an inability to perform standardized squat movements or to complete the experimental procedures properly.

For Exercise Risk Assessment, a review of each participant’s physical activity history was conducted. The Physical Activity Readiness Questionnaire (PAR-Q+) was administered to evaluate the individual’s physical condition and to ensure the safety of the testing protocol. The exercise environment was rigorously assessed, including an inspection of the venue, equipment, and protective gear, to ensure safety standards were met.

Before engaging in the study, participants received detailed information regarding the study’s objectives, methods, and potential risks, and provided their informed consent. The present study confirms that informed consent has been obtained from all participants, and the research involving human subjects is conducted in accordance with the Declaration of Helsinki; it was approved by the Ethics Committee of Zhengzhou University’s School of Basic Medical Sciences, with the reference number ZZUIRB 2023-JCYXY0016.

## Research methods

### Experimental design and process

All subjects conducted their own controlled experiment and underwent squat training with four different BFR modes at 75% 1RM exercise intensity. The BFR modes included a continuous low BFR mode (T1), an intermittent medium BFR mode (T2), an intermittent high BFR mode (T3), and a non-BFR mode (C). For T1, a continuous arterial occlusion pressure of 40% of the individual’s arterial occlusion pressure (AOP)value was applied throughout the exercise period. For group T2, AOP was consistently applied at 50% during squat exercises, with pressure relief occurring during the inter-set rest intervals to facilitate recovery. In contrast, group T3 featured 60% AOP application during the inter-set rest intervals, while the actual squat movements were performed without pressure to allow for blood flow and reduce fatigue. (C) performed squats without any compression.

**FIGURE 1:**
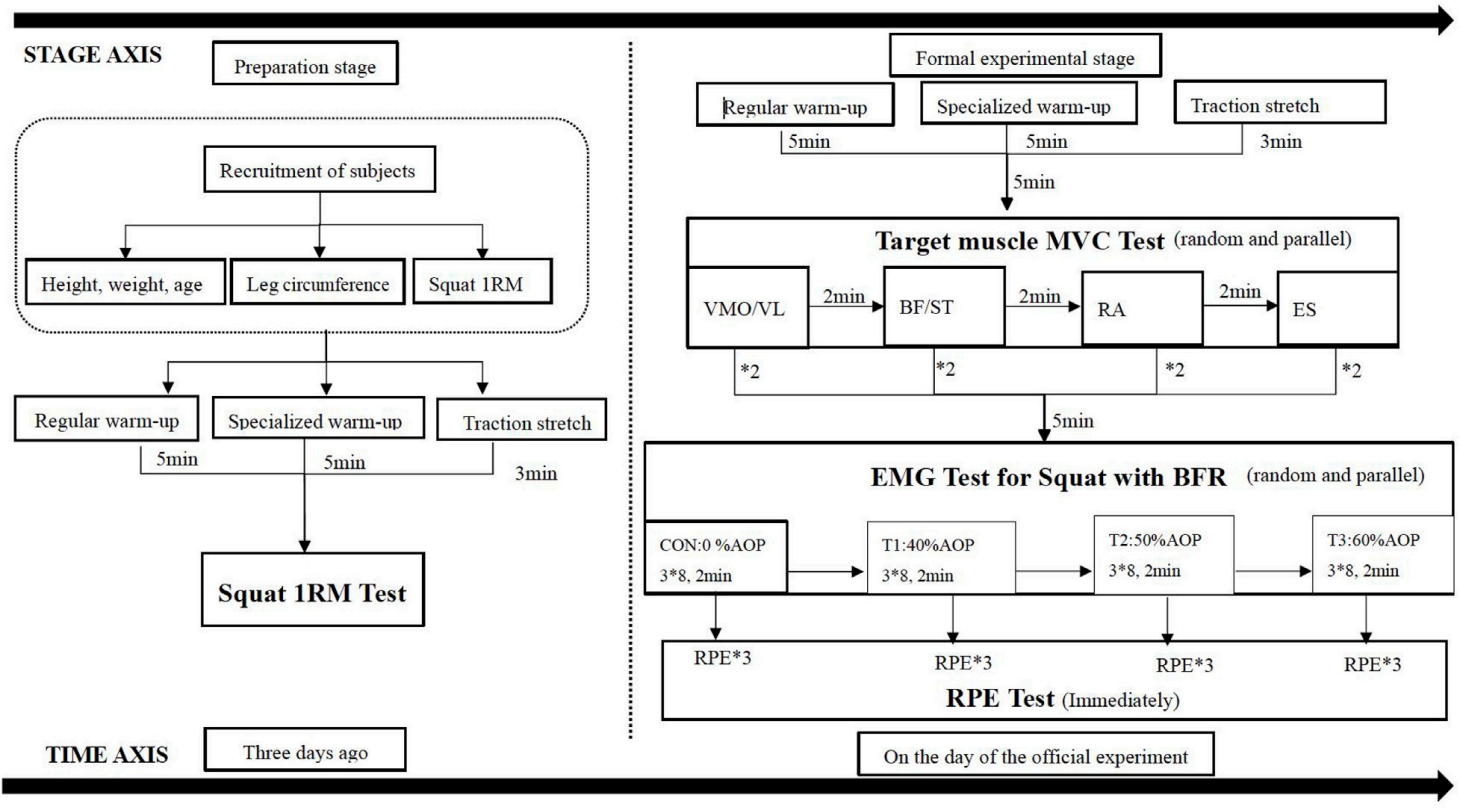
Experimental flow chart.

**FIGURE 2:**
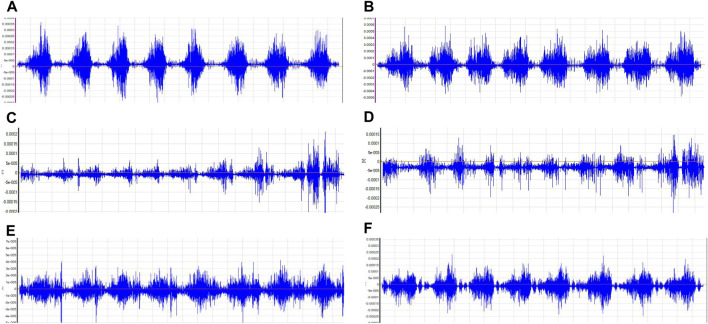
Target Muscle Group Electromyographic Illustration. Notes: **(A)**:Vastus lateralis; **(B)**:Vastus medialis obliquus; **(C)**:Biceps femoris; **(D)**:Semitendinosus; **(E)**:Rectus abdominis; **(F)**:Erector spinalis.

The resistance training scheme involved squatting with a load of 75% 1RM for three consecutive sets, with eight repetitions each set and a 2-min group interval. The sequence of squat tests under different compression modes was randomized and balanced, with a time interval of 72 h between each mode to prevent muscle damage caused by compression resistance training from affecting the test state of the subjects (Sonkodi et al., 2021) and reduce mutual interference effects between different modes. TheraBand BFR equipment was used for the intervention, applying a binding position at the root of the thigh near the proximal end with a binding pressure set at 30 mmHg (Figure 2). AOP values were calculated based on Loenneke et al.’s conversion standard “Reference Table of AOP Values Corresponding to Leg Circumference” (Loenneke et al., 2015), as shown in Table 2. The selection of low, middle, and high occlusion pressures aligned within Li Zhiyuan et al.’s range for lower limb occlusion pressures (Zhiyuan et al., 2021). The squat 1RM test was conducted 3 days before the formal experiment. On the day of the formal test, the subjects first underwent the standard warm-up procedure with the squat 1RM test and then performed the maximum voluntary contraction test of the target muscle (MVC) and the surface myoelectric test of compressed squat. Surface EMG signals were collected during both tests, leg circumference was measured immediately after each squat, and subjective fatigue was recorded, as shown in Figure 1.

**TABLE 2:** Reference table of AOP corresponding to leg circumference.

Leg circumference (cm)	60% AOP (mmHg)	50% AOP (mmHg)	40% AOP (mmHg)
<45–50.9	120	100	80
51–55.9	150	130	100
56–59.9	180	150	120
≥60	210	180	140

EMG Analysis: EMG analysis software was used to rectify, filter, smooth, and standardize the original EMG values. On the original EMG, the muscle force range was selected to cut the wave according to the start and stop time of each group of squats. The absolute value of EMG was selected for full wave rectification, and an infinite impulse response filter (IIR) was applied. The low wave was filtered at 8 Hz, and the high wave was filtered at 450 Hz (Hermens et al., 2000). Then, the root mean square (RMS) was used for smoothing processing, and the average value of RMS was taken. Finally, standardized processing was performed. The RMS value obtained in the MVC test was defined as the maximum value of the muscle force, and the average value of the muscle RMS obtained in each group of squats was divided by the RMS value during the MVC test, which is called the RMS standard value (%MVC) to reflect the degree of muscle activation (Konrad, 2005). See Formula 1 for the expression of EMG_RMS_.

$${\text{EMG}}_{\text{RMS}}=\sqrt{\frac{\sum_{i=0}^{N}Data{\left[i\right]}^{2}}{N}}$$ (1)

### Main test and observation indicators

#### 1) Squat 1RM test

**FIGURE 3:**
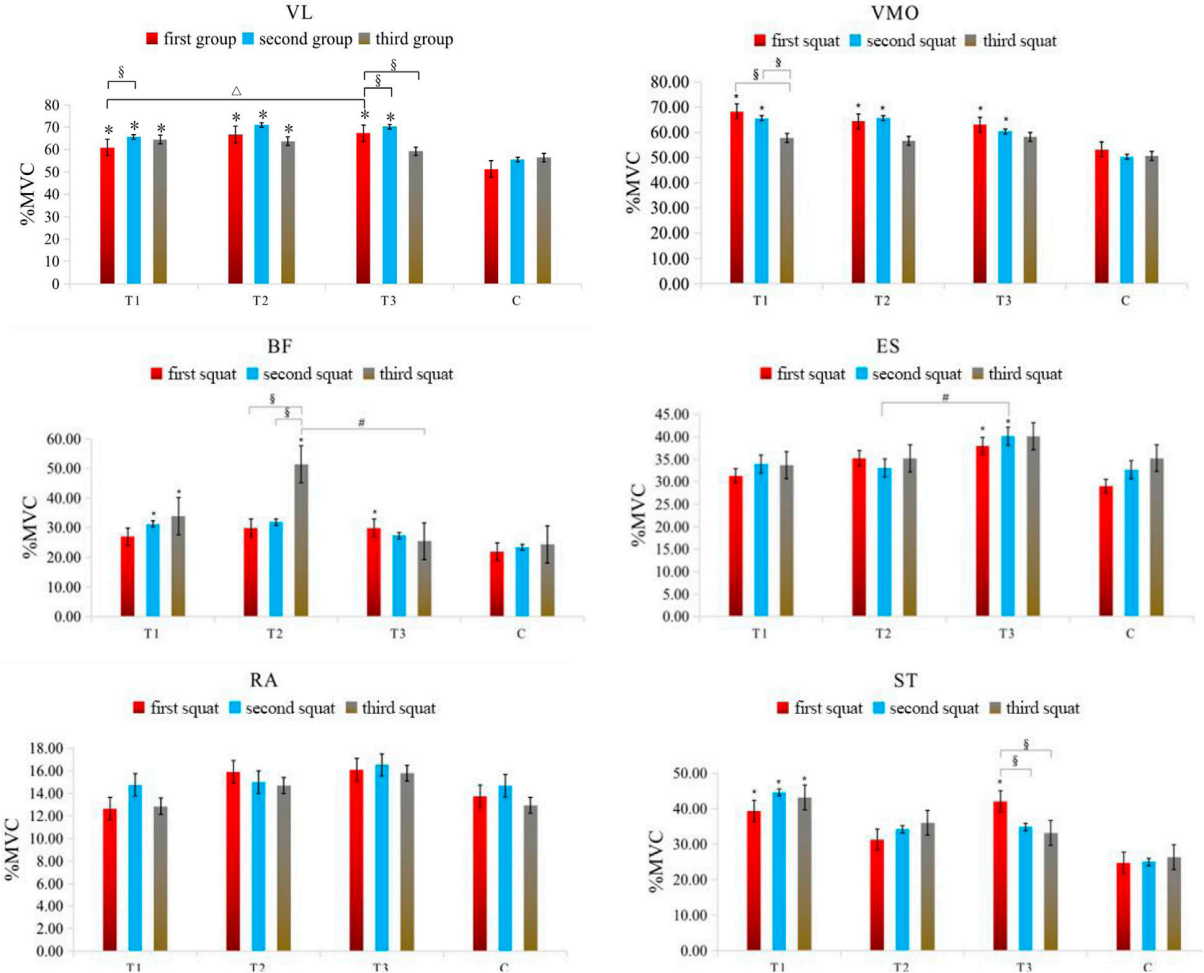
Changes in the muscle %MVC value in each group of squat training under different compression modes. Note: VL: Vastus lateralis; VMO: Vastus medialis obliquus; BF: Biceps femoris; ST: Semitendinosus; RA: Rectus abdominis; ES: Erector spinalis; * indicates a significant difference in muscle %MVC value between the pressurized experimental group and the nonpressurized blank control group; △indicates that the change in muscle %MVC value between the group T3 and group T1 is significantly different; # indicates that the change in muscle %MVC value between the group T3 and group T2 is significantly different; §indicates that there are significant differences in muscle %MVC value changes between different sets of squats in the experimental group, and the significant difference is P< 0.05.

The one-repetition maximum (1RM) squat test was administered following a standardized protocol 3 days prior to the main experiment. The standard squat technique was delineated as follows (Figure 3): participants were instructed to stand with their eyes facing forward, feet comfortably positioned outward, and hands gripping the barbell around the neck. They were then directed to perform squats in a controlled rhythm, lowering to the deepest point—defined by the hips dropping below the knees—before pausing momentarily at the base of the squat. Subsequently, they were to exert force in reverse and ascend back to the starting position with knees fully extended. In preparation for the 1RM test, subjects commenced with a standardized warm-up, which included a 5-min session at a resistance of 100 W and a step frequency of 70–80 rpm. This was followed by a 5-min period of specific warm-up exercises, involving 50% and 75% of the anticipated 1RM load. Concluding the warm-up phase was a 3-min session dedicated to muscle stretching. The 1RM test itself commenced post-warm-up, with subjects initiating the squats with a load verbally estimated at 80% of their 1RM. The load was progressively increased in increments of 4–9 kg after each set of three to five repetitions. Following a 2-min rest period, the load was incremented, and subjects performed two to three additional repetitions. This pattern continued with further 2-min rests and load increases of 4–9 kg, with an additional 2–4 kg added until the point of squat failure. The 1RM values for all subjects were ascertained across five trials, with encouragement provided to support maximal effort (Kubo et al., 2019).

#### 2) Maximum voluntary contraction (MVC) test

The Maximum Voluntary Contraction (MVC) test was initiated 5 min post-warm-up on the day of the formal experiment. Surface electromyography (EMG) signals from the target muscles were captured using a 6-channel Delsys Trigno wireless EMG acquisition system. In accordance with the biomechanics of squat movements, the following muscles were selected for analysis: for the thigh group, vastus lateralis (VL) and vastus medialis obliquus (VMO); and for the posterior thigh group, biceps femoris (BF) and semitendinosus (ST); for the core muscle, rectus abdominis (RA) and erector spinae (ES); totaling six muscle groups. Electrodes were strategically placed on the muscle bellies based on their anatomical landmarks.

Prior to electrode placement, the skin over the muscle bellies was prepared by shaving any hair and cleaning with alcohol to eliminate any dust or sweat, thereby reducing skin impedance and ensuring optimal sensor contact. MVC test data were collected for each muscle group following Konrad’s protocol (Konrad, 2005), which involved two MVC tests per muscle. The test procedures were as follows:

1. RA: Subjects were positioned supine with the knees flexed at 30°, holding their ankles and performing a maximal voluntary contraction of the upper body while the tester applied downward resistance on the chest for 3–5 s (left panel).
2. ES: Subjects lay prone with the lower limbs secured, and maximal force was applied to the scapula while the tester provided resistance for 3–5 s (right panel).
3. VMO and VL: With the subject seated and the torso and thigh at 90-degree angles, the knee was fully extended. The tester applied downward resistance at the ankle’s upper end for 3–5 s (left panel).
4. BF and ST: Subjects were positioned prone with the right knee joint bent to approximately 20° in a “C” shape. Downward resistance was applied to the upper ankle, maintained for 3–5 s, and the EMG data were collected (right panel).

#### 3) Surface EMG test of pressurized squat

After completing the MVC test, the subject rested for 5 min, and then the tester prepared the subsequent pressurized squat surface EMG test by binding the subject. The subject followed the standard squat movements for three squat exercises. Before each squat, the camera and EMG acquisition system were turned on in preparation. Once the subject began the squat, the acquisition system began recording the EMG signal data. Based on the experimental synchronous video recording, all EMGs from the first to the third squat of each pressurized squat session were selected for subsequent data analysis.

#### 4) Thigh circumference test

The thigh circumference test was conducted in the preparation stage 3 days before the experiment and immediately after each group of pressure squat training in the formal experiment. Subjects stood with their legs shoulder-width apart, and a tape measure was placed horizontally on the transverse line below the rear hip to measure the thigh circumference. Each leg’s circumference was measured three times and averaged (Zhiyuan et al., 2021).

#### 5) RPE test

Prior to the experimental procedures, participants were thoroughly briefed on the rating scale to be utilized for assessing perceived exertion. Following each session of squat training across varying BFR modalities, subjects were required to self-rate their perceived exertion, which was subsequently documented. The RPE scale, as adapted by Zourdos et al. (2021), was employed in this study. This scale, tailored for evaluating the subjective fatigue levels during resistance training, ranges from 1 to 10, with the initial four levels being based on the subjective assessment of effort. Specifically, levels 1–2 denote no effort, while levels 3–4 indicate a minimal degree of exertion. The subsequent levels, 5–10, are determined by the number of repetitions in reserve (RIR): levels 5–6 suggest the capacity to perform an additional 4-6 squats post-training; level 7 corresponds to three more repetitions; level 8 to two more repetitions; level 9 to one additional repetition; and level 10 signifies maximum effort.

### Statistical analysis

Statistical analysis of the electromyography (EMG) data was conducted using Excel 2010 and SPSS 17.0 software following the aforementioned procedures. Data were presented as the mean ± standard deviation (M±SD). Two-factor repeated measures analysis of variance (ANOVA) (BFR mode × exercise sets) was utilized to analyze the RMS standard values (%MVC) of the target muscles of the lower limbs and core muscles in different subjects under various compression modes of high-intensity squat training. Before conducting the ANOVA, Mauchly’s spherical test was performed to assess the sphericity assumption. If the test result was p > 0.05, aligning with the Huynh-Feldt conditions, we accepted the results of the spherical hypothesis test and proceeded with one-way ANOVA; if the test result was p < 0.05, indicating a violation of the sphericity assumption, we applied the Greenhouse-Geisser correction to adjust the degrees of freedom. Upon completion of repetitive measure ANOVA, multiple comparisons to adjust may cause risk of the first type of mistake, we have adopted Tukey HSD (Honestly Significant Difference) test. This method was adapted to our study design and allowed for simultaneous adjustment for group comparisons. Specifically, where ANOVA subject or interaction effects were found to be significant, Tukey HSD tests were used to determine which group differences were statistically significant. We followed standard statistical procedures and adjusted the P-value for each pair of comparisons, ensuring statistical correction and reliability of the results.

## Results

A subject’s raw EMG of various muscle groups during squat with BFR training (Figure 2).

### Results of two-factor analysis of variance

Two-factor repeated-measures ANOVA revealed that BFR mode had a significant effect on the %MVC values of VL (F = 20.175, p = 0.000), VMO (F = 9.267, p = 0.000), BF (F = 4.021, p = 0.041), ST (F = 8.437, p = 0.000), and ES (F = 5.593, p = 0.002) (p < 0.05); the number of exercise sets significantly influenced the %MVC values of VL (F = 4.956, p = 0.018) and VMO (F = 6.430, p = 0.006) (p < 0.05); the interaction between different BFR modes and the number of exercise sets was not statistically significant for the %MVC values of all muscles (p > 0.05). It can be seen that different pressurization mode interventions are the main factors affecting the degree of muscle activation change during high-intensity squat training. The specific results are shown in Table 3.

**TABLE 3:** Effects of BFR mode, exercise group and their interaction on target muscle %MVC.

	Anterior thigh muscles				Back side muscles in the thigh				Core muscle			
	VL		VMO		BF		ST		RA		ES	
	F	P	F	P	F	P	F	P	F	P	F	P
BFR mode	20.175	0.000*	9.267	0.000*	4.021	0.041*	8.437	0.000*	0.424	0.608	5.593	0.002*
Exercise sets	4.956	0.018*	6.430	0.006*	3.001	0.083	0.028	0.973	0.360	0.659	3.155	0.063
Interaction groups	2.242	0.071	0.487	0.816	1.260	0.301	0.667	0.667	0.037	0.992	0.391	0.883

Notes: VL: vastus lateralis; VMO: vastus medialis obliquus; BF: biceps femoris; ST: semitendinosus; RA: rectus abdominis; ES: Erector spinalis.* indicates a significant difference, with a p-value <0.05.

### Changes in the muscle %MVC value in each group of squat training under different compression modes

In the anterior thigh muscles, the %MVC of vastus lateralis percentages for groups T1, T2, and T3 were significantly higher than group C during the first squat (p < 0.05), with T3 showing a higher value than T1 (p = 0.032). During the second squat, the MVC percentages for T1, T2, and T3 remained significantly higher than C. In the third squat, only groups T1 and T2 maintained a significant MVC percentage increase over C. Additionally, T1 showed a significant increase in MVC percentage from the first to the second squat (p = 0.047), while T3 showed significant increases in MVC percentage during the first and second squats compared to the third (p = 0.014 and p = 0.009, respectively). For the vastus medialis, in the first squat, the MVC percentages for T1, T2, and T3 were significantly higher than C (p < 0.05). In the second squat, the MVC percentages for T1, T2, and T3 continued to be significantly higher than C. Furthermore, T1 showed significant increases in MVC percentage from the third squat compared to both the first (p = 0.025) and second squats (p = 0.027).

In the posterior thigh muscles, specifically the biceps femoris, T3 had a significantly higher MVC percentage than C in the first squat (p = 0.039), and T1 in the second squat (p = 0.040). In the third squat, both T1 and T2 had significantly higher MVC percentages than C, with T2 also showing a significant difference compared to T3 (p = 0.020). Moreover, T2 showed significant increases in MVC percentage during the third squat compared to the first (p = 0.005) and second squats (p = 0.006). For the semitendinosus, T1 and T3 had significantly higher MVC percentages than C in the first squat (p < 0.05), with only T1 maintaining this difference in the second and third squats (p < 0.05). Additionally, T3 showed significant increases in MVC percentage during the first squat compared to the second (p = 0.039) and third squats (p = 0.009).

Core muscle group: Rectus abdominis: There were no significant differences in the %MVC value of rectus abdominis among all groups (P> 0.05). Erector spine muscle: In the first squat, the %MVC value of the group T3 (p = 0.011) was significantly higher than that of the C group (P< 0.05). In the second squat, the %MVC value of the group T3 (p = 0.030) was also significantly higher than that of the C group (P< 0.05), at the same time, the activation degree of group T3 (p = 0.023) was significantly higher than that of group T2 (p < 0.05). The specific results are shown in Table 4 and Figure 3.

**TABLE 4:** Different BFR modes squats targeted muscle in training the MVC %.

Muscle	BFR	Three sets squat (8 + 8+8)			ES (95%CI)			F	η^2^
		First set	Second set	Third set	T1	T2	T3		
VL	C	51.29 ± 8.44	55.45 ± 11.67	56.47 ± 12.26	−0.73[−1.0, −0.47]	0.13[−0.11,0.38]	0.82[0.51,1.2]	20.175	0.428
	T1	60.91 ± 14.25*^△^	65.59 ± 15.42*^§^	64.46 ± 13.41*	∼	0.24[−0.01,0.51]	0.13[−0.11,0.38]		
	T2	66.70 ± 11.40*	71.06 ± 13.47*	63.72 ± 18.89*	∼	∼	−0.1[−0.32,0.12]		
	T3	67.34 ± 14.20*^§^	70.26 ± 13.68*^§^	59.27 ± 18.34	∼	∼	∼		
VMO	C	53.12 ± 9.47	50.29 ± 13.81	50.71 ± 13.06	1.14[0.72, 1.65]	0.70[0.28, 1.17]	0.67[0.35, 1.05]	9.267	0.256
	T1	68.22 ± 8.68*^§^	65.53 ± 8.82*^§^	57.79 ± 8.87	∼	−0.17[−0.59,0.24]	−0.26[−0.64,0.09]		
	T2	64.37 ± 17.18*	65.72 ± 17.26*	56.65 ± 15.79	∼	∼	−0.07[−0.33,0.19]		
	T3	63.01 ± 14.26*	60.39 ± 13.44*	58.15 ± 17.33	∼	∼	∼		
BF	C	21.91 ± 7.88	23.38 ± 12.13	24.40 ± 10.03	0.62[0.29, 1.00]	0.54[0.16, 0.96]	0.44[0.04, 0.88]	4.021	0.13
	T1	26.97 ± 12.97	31.29 ± 14.44*	33.82 ± 13.76*	∼	0.25[−0.02,0.54]	−0.31[−0.77, 0.13]		
	T2	29.86 ± 16.21^§^	31.88 ± 20.14^§^	51.35 ± 56.67*	∼	∼	0.40[0.01,0.82]		
	T3	29.90 ± 9.62*	27.32 ± 6.78	25.41 ± 7041^#^	∼	∼	∼		
ST	C	24.68 ± 10.04	25.02 ± 9.55	26.32 ± 9.45	1.06[0.63,1.57]	0.59[0.15,1.07]	0.75[0.39,1.16]	8.437	0.238
	T1	39.37 ± 19.34*	44.54 ± 22.81*	43.14 ± 20.76*	∼	−0.44[−0.81, −0.1]	−0.29[−0.62,0.03]		
	T2	31.19 ± 17.06	34.21 ± 21.85	35.98 ± 15.57			−0.15[−0.61,0.29]		
	T3	42.01 ± 20.86*	34.81 ± 20.21^§^	33.16 ± 16.25^§^	∼	∼	∼		
RA	C	13.77 ± 3.06	14.72 ± 3.06	12.96 ± 3.06	−0.04[−0.46,0.38]	0.08[−0.17,0.34]	0.15[−0.08,0.4]	0.424	0.015
	T1	12.65 ± 3.67	14.75 ± 3.67	12.87 ± 3.67	∼	0.1[−0.27,0.48]	0.17[−0.21,0.57]		
	T2	15.92 ± 7.15	15.02 ± 7.15	14.70 ± 7.15	∼	∼	0.05[−0.04,0.13]		
	T3	16.13 ± 6.21	16.54 ± 6.21	15.80 ± 6.21	∼	∼	∼		
ES	C	29.04 ± 4.23	32.66 ± 4.23	35.26 ± 4.23	0.03[−0.24,0.3]	0.16[−0.15,0.47]	0.51[0.25,0.8]	5.593	0.172
	T1	31.35 ± 4.83	33.94 ± 4.83	33.70 ± 4.83	∼	0.12[−0.15,0.4]	0.46[0.19,0.76]		
	T2	35.28 ± 4.91	33.03 ± 4.91#	35.22 ± 4.91	∼	∼	−0.33[−0.57, x−0.11]		
	T3	38.03 ± 4.87*	40.13 ± 4.87*	40.11 ± 4.87	∼	∼	∼		

### Changes in thigh circumference in each group of squat training under different BFR modes

The left leg circumference of the group C (p = 0.007), group T1 (p = 0.001), group T2 (p = 0.001) and group T3 (p = 0.005) after training was significantly higher than that measured before training. The right leg girth of the C group (p = 0.001), group T1 (p = 0.002), group T2 (p = 0.003) and group T3 (p = 0.000) after training was significantly higher than that of the right leg girth measured before training (P< 0.05). The results are shown in Table 5.

**TABLE 5:** Changes in hindleg circumference in each group of squat training under different pressure modes.

		Left leg circumference (cm)	Right leg circumference (cm)
pre-test	C/T1/T2/T3	56.06 ± 1.77	55.75 ± 2.29
post-test	C	56.73 ± 1.70*	56.67 ± 2.25*
	T1	57.24 ± 2.12*	57.17 ± 1.90*
	T2	57.49 ± 2.27*	57.45 ± 2.27*
	T3	57.17 ± 2.57*	57.06 ± 2.39*

Note: * indicates that there is a significant difference in the changes in leg circumference between the post-test and pre-test leg circumference after exercise intervention with different pressure resistance modes; The significant difference is P< 0.05.

### Test results of subjective fatigue after squat training in each group under different BFR modes

The RPE value of the group T2 after the first squat (p = 0.013), the second squat (p = 0.005) and the third squat (p = 0.007) was significantly higher than that of the blank control group C. The RPE value of the group T1 during the third squat (p = 0.011) was significantly higher than that of the blank control group C (P< 0.05). At the same time, the RPE value of the nonpressurized blank control group C during the second (p = 0.024) and third (p = 0.004) squats was significantly higher than that of the first squats. The RPE value of the third squat in the group T1 was significantly higher than that of the second squat (p = 0.037) and the first squat (p = 0.001), and the RPE value of the second squat was also significantly higher than that of the first squat (p = 0.005). The RPE values of the second (p = 0.005) and third squats (p = 0.011) in the group T2 were also significantly higher than those of the first squats (P< 0.05). In contrast, there was no significant difference in RPE values in the group T3 during all three squats (P> 0.05). The specific results are shown in Table 6.

**TABLE 6:** List of changes in subjective fatigue after squat training in each group under different pressure modes.

	BFR mode	Exercise sets		
		First	second	Third
RPE value	C	5.2 ± 1.14	5.8 ± 1.40^§^	6.1 ± 1.50^§^
	T1	5.9 ± 1.28	6.5 ± 1.51^§^	6.9 ± 1.29*^§#^
	T2	6.4 ± 1.78*	7.0 ± 1.89*^§^	7.2 ± 1.55*^§^
	T3	6.1 ± 1.10	6.6 ± 1.17	6.9 ± 1.37

Note: * indicates a significant difference in RPE, values between the pressurized experimental group and the non-pressurized blank group C; §indicates that there are significant differences in RPE, values between the first squats and the second and third squats in each experimental group; # indicates that there are significant differences in RPE, values between the second squat and the third squat; the significant difference is p < 0.05.

## Discussion

### Analysis of changes in the degree of muscle activation in the anterior thigh muscles

The present study’s findings demonstrate that the percentage of %MVC for anterior thigh muscles during the initial two sets of squats under diverse compression conditions was markedly elevated in comparison to the non-compressed state, corroborating the outcomes of prior investigations. In a 2021 study, Che Tongtong et al. facilitated four sessions of half-squat compression training for female wrestlers at a pressure of 180 mmHg and an exercise intensity of 30% 1RM, noting a significantly higher muscle activation level in the experimental group subjected to compression compared to the non-compressed control group (Tong-Tong et al., 2021). Consistent with these findings, Li Zhiyuan et al. (2021) observed that both continuous and intermittent pressures, ranging from 40% to 60% AOP, significantly augmented the activation level of Vastus lateralis and Vastus medialis in handball players during low-intensity (30% 1RM) squat training (Zhiyuan et al., 2021). It has been established that pressure stimulation can enhance the reliance on anaerobic metabolism for energy provision (Manini et al., 2011), causing rapid exhaustion of type I muscle fibers and a subsequent increase in the recruitment of type II muscle fibers with higher activation thresholds. Furthermore, the application of pressure during muscle contractions induces intramuscular hypoxia due to restricted blood flow, resulting in the accumulation of metabolic byproducts, such as lactic acid. This accumulation leads to an “overload” effect, triggering a cascade of stress responses that activate the III and IV afferent nerve centers (Yasuda et al., 2006). These responses, in turn, stimulate muscle fiber recruitment and may potentially inhibit α motor neurons (Yasuda et al., 2006). As a result, a greater number of muscle fibers are recruited to sustain equivalent force and mechanical power output, as evidenced by the observed significant increase in muscle activation (Gizzi et al., 2021).

Significantly, the activation level of the lateral femoral muscle during the initial squat session was observed to be higher under high-pressure conditions group T3 than under medium-pressure conditions group T2, a result that aligns with prior research. Zhang and Yi (2022) conducted high-intensity (75% 1RM) squat training with subjects under very high pressure and reported increased muscle activation during the eccentric phase of squats under high pressure (350 mmHg) as opposed to low pressure (250 mmHg). A synthesis of the dose-response relationship in BFR (Lin et al., 2023) suggests an “inverted U-shaped” correlation between pressure and muscle functional performance. Within an optimal pressure range, incremental occlusion pressure intensifies the blood flow blockade and augments the stimulation effect, culminating in peak stress and adaptation responses at a critical pressure value, which is conducive to achieving the best training outcomes. Conversely, pressures surpassing this threshold can lead to a decline in training efficacy due to excessive metabolic load that overwhelms the body’s adaptive capacity. The findings imply that a 60% arterial occlusion pressure during high-intensity BFR squat training is within the effective pressure range for positive stimulation. Additionally, the activation level of the lateral femoris muscle during the second squat under continuous low-pressure conditions group T1 was notably higher than during the first, potentially attributable to the post-activation potentiation (PAP) effect. Moore et al. (2004) and Wilk et al. (2020) have successively demonstrated that unilateral elbow flexion training and bench press training of the upper limbs, combined with BFR intervention, can induce the occurrence of PAP effects in the upper limbs, significantly enhancing the activation capacity of the upper limb muscles and promoting exercise performance. Furthermore, Doma et al. (2020) and Hongwen and Xiang (2022) have found in subsequent studies that lower limb training combined with BFR intervention can also significantly induce PAP effects in the lower limbs, significantly improving lower limb explosive power and enhancing the performance of lower limb muscle movements. This effect is mediated by the recruitment of high-threshold motor units, which elevates the phosphorylation of light chains, enhances the sensitivity of calcium ions (Ca2+) within muscle cells, and thus improves muscle fiber contractility, leading to enhanced exercise performance. As a result, the power output and motor performance in subsequent training movements surpass those of the initial movements (Lin et al., 2023).

### Analysis of changes in the degree of muscle activation in the thigh posterior group

The study’s findings indicate that under the continuous low-pressure BFR mode group T1, there was a significant increase in the activation levels of the biceps femoris and semitendinosus muscles during the second and third sets of squats. In comparison, the intermittent medium-pressure BFR mode group T2 showed an increase in activation only during the third set, and the high-pressure BFR mode group T3 demonstrated an increase solely in the first set. Biomechanically, a complete squat is a complex, multi-joint movement that requires the coordinated action of muscles across the hip, knee, and ankle joints, with different muscle groups assuming specific roles in force transfer and joint movement (Trujillo et al., 2011). The muscles in question are antagonists, and their increased activation under the group T1 condition in the latter sets suggests a progressive recruitment pattern. According to the principles of motor unit recruitment, as the load intensity and volume of an exercise reach certain thresholds, there is a corresponding increase in the activation of both agonist and antagonist muscle groups involved in the movement. Thus, the study’s outcomes are consistent with established muscle recruitment dynamics.

Among the BFR modes tested, only the continuous low-pressure mode group T1 was found to consistently and effectively enhance the activation of the posterior muscle group. The other two intermittent pressure modes showed variable effects on muscle activation levels. Upon comparison, the intermittent release of pressure likely reduced the tissue hypoxia and excessive metabolic stress associated with continuous BFR, leading to less pronounced muscle activation increases. The intermittent modes’ approach to pressure application may not have provided sufficient metabolic stress to further enhance muscle activation beyond a certain point. However, the continuous low-pressure mode allows for a cumulative metabolic stress that improves muscle fiber recruitment efficiency and contractile capacity, which can be advantageous for squat performance. Consequently, the study suggests that utilizing a continuous low-pressure BFR mode may be more beneficial for squat training outcomes.

### Analysis of changes in the muscle activation degree of the core muscle group

According to the study (Sha and Luo, 2015), the effective activation of a series of core muscles, such as the rectus abdominis muscle and erector spinae muscle, is crucial for maintaining movement stability during squats, enhancing the overall success of the subjects’ transition from squatting to standing. The findings of this study indicated that there was no significant difference in the activation level of the rectus abdominis muscle regardless of whether compression intervention was applied, which contrasts with previous research. Tong-Tong et al. (2021) suggested that continuous compression intervention during low-intensity half-squat training could lead to the transfer of training effects, with the activation level of the rectus abdominis in the non-compression area also significantly improved (Tong-Tong et al., 2021). This discrepancy in results can be attributed to differences in training intensity, training movements, and individual characteristics. In this study, college students were recruited to participate in the experiment, and the resistance training protocol involved compressive squat training at 75% 1RM exercise intensity. The mechanical load pressure and metabolic pressure of this training far exceeded those in the previous study, while the training level of the college students may have been lower, resulting in a higher overall difficulty in completing the movements. During the training process, the activation level of the rectus abdominis muscle consistently peaked during exercise, so there was no significant change in the activation level of this muscle regardless of whether pressure intervention was applied.

The activation level of the erector spinae muscle during the first and second squats was significantly higher in the group T3 condition compared to the control group; that is, the intermittent high-pressure compression mode more effectively enhanced the activation level of the erector spinae muscle compared to the other modes. This may be related to the higher blocking pressure and sufficient intermittent rest. On one hand, higher blocking pressure can induce greater metabolic stress stimulation and promote the recruitment of fast muscle fibers (Gizzi et al., 2021). On the other hand, after pressure relief, some metabolic pressure can be reduced to ensure rapid synthesis of phosphocreatine, thereby minimizing acidosis caused by the accumulation of metabolic products and muscle fatigue resulting from insufficient energy supply. This allows for the avoidance of exercise fatigue (Zhiyuan et al., 2021) after the release of the pressure cuff and restoration of neural regulation of muscle function.

### Analysis of changes in leg circumference test results

The study’s results demonstrate that squat training, irrespective of pressure stimulation, led to a significant increase in leg circumference. Post-training measurements revealed that the circumference of both the left and right legs was notably higher than pre-training measurements across all groups. Prior research (Joyner and Casey, 2015) has established that exercise is a potent activator of the autonomic nervous system, potentially causing muscle congestion. The muscular contractions during exercise elevate central hemodynamics, triggering a cascade of autonomic responses. This includes increased cardiac output, ventilation, vascular sympathetic tone, and blood flow to the active muscles, which collectively contribute to muscle congestion. This physiological response may account for the observed increase in leg circumference following acute exercise, regardless of whether compression was applied.

However, a divergence from some scholarly observations (Loenneke and Pujol, 2009) was noted; typically, a more pronounced increase in limb circumference is expected in the pressurized group post-exercise compared to the non-pressurized group. The rationale provided is that pressurization may impede venous return, leading to limb effusion and subsequent reactive congestion. The pressure gradient created by pressurization may facilitate the shift of effusion from the plasma into muscle cells. Contrary to these findings, the current study’s results suggest that the muscle congestion may have already peaked due to the high-intensity nature of the resistance training, implying that additional pressure stimulation did not surpass the threshold for further increasing muscle congestion.

### Analysis of changes in subjective fatigue test results

RPE, as utilized by the Center for Sports Training, serves as a critical tool for gauging mental fatigue during athletic endeavors and for adjusting exercise intensity in real-time (Tong-Tong et al., 2021). The current study’s findings suggest that both continuous low-pressure and intermittent medium-pressure BFR can lead to increased psychological fatigue among participants. However, the intermittent high-pressure BFR mode was associated with a more favorable subjective fatigue perception. Previous research by Vieiea et al. (2015), Schwiete et al. (2021), and Tong-Tong et al. (2021) has consistently shown that in controlled trials, the RPE is significantly higher in the pressurized group compared to the non-pressurized group, likely due to the elevated metabolic stress from pressurization. Notably, the present study observed no significant difference in RPE values across the different BFR modes.

This discrepancy may stem from the fact that previous studies altered only the BFR mode while maintaining a constant pressure level. In contrast, the current study featured variations in both the BFR mode and the pressure intensity, with the continuous mode at a low pressure and the intermittent modes at medium and high pressures. It is hypothesized that the intermittent mode, despite allowing for recovery periods, may not reduce the RPE value significantly due to the higher metabolic demand, resulting in similar RPE values to the continuous mode. Strikingly, the intermittent high-pressure BFR mode yielded the lowest RPE values, suggesting that the asynchronous application of mechanical load and metabolic stress during intermittent pressurization does not accumulate fatigue to the same extent as continuous pressure. Consequently, participants reported a lower subjective fatigue level, indicating that this mode may be particularly suitable for high-resistance training in a collegiate setting. Furthermore, the study observed a progressive increase in RPE values across the three sets of squat training, with significant elevations from the first to the second and third sets. This increase can be attributed to the physiological consequences of continuous resistance training, including muscular and nervous fatigue, depletion of energy resources, and the accumulation of metabolic byproducts, all of which contribute to a heightened sense of physical and psychological fatigue.

## Conclusion

In the realm of BFR and squat exercises, all BFR modalities have been found to significantly enhance the activation of the anterior thigh muscles. Continuous low-pressure BFR uniquely and reliably boosts the activation of the posterior thigh muscles, while intermittent modes show variable effects. Although BFR does not alter leg circumference, it does elevate subjective fatigue levels. Notably, the intermittent high-pressure BFR mode is particularly effective at enhancing erector spinae activation and produces the least subjective fatigue during squats. Given the overall effectiveness and practicality of training, the intermittent high-pressure BFR mode stands out as advantageous for improving muscle activation and fostering neuromuscular adaptations, which are key objectives in training regimens.

## Data Availability

The raw data supporting the conclusions of this article will be made available by the authors, without undue reservation.
